# Learning Curve Associated With Operative Treatment of Terrible Triad Elbow Fracture Dislocations

**DOI:** 10.7759/cureus.27156

**Published:** 2022-07-22

**Authors:** Yagiz Ozdag, A. Michael Luciani, Stephanie Delma, Jessica L Baylor, Brian K Foster, Louis C Grandizio

**Affiliations:** 1 Orthopaedics, Geisinger Medical Center, Danville, USA; 2 Orthopaedic Surgery, Geisinger Medical Center, Danville, USA; 3 Orthopaedics, Geisinger Commonwealth School of Medicine, Scranton, USA

**Keywords:** surgical complications, elbow trauma, upper-extremity trauma, terrible triad, complex elbow dislocation

## Abstract

Purpose: To assess the outcomes of operatively treated terrible triad (TT) elbow injuries for a single surgeon at the start of clinical practice. We aimed to define postoperative patient reported outcome measures (PROMs), range of motion (ROM), and complications during the period immediately following fellowship training, in order to describe the learning process for surgical treatment of TT.

Methods: All operatively treated TTs from 2017 to 2020 were included. All cases were performed by a single, fellowship-trained upper-extremity surgeon and represented a consecutive series at the start of clinical practice. Baseline demographics, injury characteristics, and surgical details were recorded for each case. PROMs [QuickDisability of arm, shoulder, and hand (DASH) and (visual analog scale) pain scale], ROM, and complications were recorded at the time of final follow-up. A perioperative glucocorticoid protocol was used in all cases without diabetes.

Results: There was a total of 21 included TT cases with a mean follow-up of 20 months. The operative time averaged 89 min for the first 10 cases and 83 min for the subsequent 11 cases. The mean QuickDASH and VAS pain score at final follow-up were 19 and 2.3, respectively. The mean flexion-extension arc was 122° and two cases (9%) had < 100° arc of motion. The mean pronation-supination arc was 145°. Three cases (14%) had a postoperative complication, all of which underwent reoperation. Of the 21 included cases, these reoperations represented cases #1, #14, and #17 respectively.

Conclusions: Upper-extremity surgeons at the start of clinical practice may be able to achieve outcomes similar to more experienced surgeons for operatively treated TT elbow fracture dislocations. There does not appear to be a substantial “learning curve” after fellowship training with respect to PROMs, complication rates, or operative time associated with surgical treatment of TT elbow injuries.

## Introduction

In the elbow, the “terrible triad” (TT) refers to a complex injury involving dislocation of the ulnohumeral joint with fractures of the coronoid and radial head [1]. These complex elbow fracture dislocations can result from both high- or low-energy mechanisms and often involve a fall on an outstretched hand [1]. These injuries have historically resulted in poor functional outcomes and high rates of surgical complications, often due to recurrent instability and elbow stiffness [1-3]. More recent standardized surgical protocols focusing on early operative intervention, stable osseous fixation, and ligament repair have resulted in improved outcomes and decreases in complication rates [4-6]. Even with efforts to standardize surgical treatment, there remains controversy regarding methods of ligament repair and reconstruction, the need for coronoid fracture fixation, and the timing of postoperative mobilization. 

Despite improvements in operative techniques and outcomes, TT surgical procedures remain technically challenging. This may be particularly evident for surgeons at the start of their clinical practice. There has been a recent emphasis on the relationship between early surgeon experience and surgical outcomes. Prior investigations have aimed to analyze practice patterns of early career upper-extremity surgeons as well as assess surgical complications for surgeons within their board collection period [7-8]. Studies involving shoulder arthroplasty procedures performed by early career surgeons have demonstrated decreased operative times, lower complication rates, and overall decreases in healthcare costs as surgeons gain more experience [9-10]. While these investigations suggest there may be a “learning curve” associated with some procedures, prior studies assessing elbow arthroscopy suggest that a complication-based learning curve may not exist [11]. Understanding if a particular number of cases is required to reach a level of competency may be an important consideration, as the concept of “minimum-volume standards” has been implemented at various institutions for a number of surgical specialties in an effort to limit allowable procedures based on the surgeon or hospital experience [12]. 

At present, there is a paucity of prior investigations assessing outcomes of TT procedures relative to surgeon experience and it remains unknown how many cases are required to reach a level of proficiency. The purpose of this investigation was to assess the outcomes of operatively treated TT elbow injuries for a single surgeon at the start of clinical practice. We aimed to define postoperative patient reported outcome measures (PROMs), range of motion (ROM), and complications during the period immediately following fellowship training, in order to describe the learning process for surgical treatment of TT. We hypothesized that with modern treatment protocols and techniques, surgical outcomes would be comparable to recently published series and systematic reviews.

## Materials and methods

Institutional review board approval was obtained for this retrospective investigation. All data were obtained from a prospectively managed elbow trauma database. All cases involving operative treatment of a TT elbow fracture dislocation in patients 18 years of age or older were included from August 2017 to December 2020. We excluded cases with <6 months of clinical follow-up. All cases were performed by a single, fellowship-trained hand and upper-extremity surgeon. Cases were performed within our rural, Level I trauma center, which is part of an integrated, academic, tertiary referral center in the northeastern United States. The treating surgeon started practice in August of 2017 and this case series represented a consecutively treated series of patients at the start of clinical practice. 

After identifying included TT cases, a manual chart review was performed. Baseline demographics were recorded, and assessments were performed on a per-case (rather than per-patient basis) to account for patients with bilateral injuries. Demographics included age, sex, laterality, medical comorbidities as well as the presence of any mental, behavioral or neurodevelopmental disorders, which included any ICD-10 codes from F01 to F99. Injury characteristics were recorded for each case including mechanism and associated injuries. Radial head fractures were described using the Broberg-Morrey modification of the Mason classification system [13]. Coronoid fractures were described using the Regan and Morrey Classification [14].

The operative report, intraoperative, and postoperative radiographs were reviewed to record details of the surgical procedure. We recorded operative time (defined as the time in minutes from incision to the completion of skin closure) and surgical details for the TT procedures. PROMs, which included QuickDASH (disability of the arm, shoulder, and hand) and visual analog scale (VAS) for pain, were recorded as well as ROM measurements at the time of final follow-up. All ROM measurements were measured and recorded by a certified occupational hand therapist using a manual goniometer. 

Surgical details

Operative treatment of TT injuries generally followed the modified Pugh protocol [5-6]. After induction of general anesthesia, patients were positioned supine on a hand table. A sterile brachial tourniquet was used for hemostasis and a local anesthetic was used in line with the posterior incision on the elbow. A postoperative peripheral nerve block was utilized to allow for neurovascular assessment immediately after the procedure. A lateral subcutaneous flap was elevated to expose the extensor-supinator muscles. In cases where a lateral collateral ligament (LCL) repair was expected to be performed, the extensor digitorum communis (EDC)-split approach was utilized. In cases where an LCL repair was augmented with a braided non-absorbable suture-tape (internal brace), Kocher’s interval was used. The radiocapitellar joint capsule was opened and both the radial head and capitellum were visualized. Care was taken to protect the posterior interosseous nerve distally. 

Next, the radial head was inspected. Radial head fractures with three or more fragments that were not amenable to fixation underwent excision for subsequent cementless, metallic, and radial head arthroplasty. Otherwise, open reduction, internal fixation (ORIF) was performed with headless compression screws. In two cases with small (<10%) articular fragments, partial excision was performed. Fixation of the coronoid fracture (when performed) was done before addressing the radial head to allow for improved visualization. Coronoid fracture fixation was performed at the surgeon’s discretion. As described by previous authors, in cases where the elbow was felt to be stable by addressing the radial head and LCL, the coronoid was not routinely fixed [15]. When performed, the coronoid and anterior capsule was repaired utilizing the trans-osseous suture lasso technique for both type I and type II fractures [16].

Next, the LCL complex was addressed. The senior author performed LCL repair with suture-tape augmentation (as opposed to repair alone) in three situations: TT injuries associated with a high-energy mechanism, a “bald” lateral condyle with avulsion of LCL and tendon origins, and cases where there was a re-dislocation in the splint after reduction in the emergency department. LCL repairs were performed with a #2, braided, non-absorbable suture placed in a running-locking fashion along the course of the LUCL and docked into a 4.75-mm SwiveLock (Arthrex, Naples, FL) anchor in the anatomic origin of the LCL on the humerus. LCL repair with suture-tape augmentation utilized the same anchor at the anatomic LCL origin on the lateral condyle and at the supinator crest of the ulna (LCL insertion), in a manner similar to that described by Greiner et al. [17]. Repair of the native LCL was performed over the suture tape with #2, braided, non-absorbable sutures. 

After stabilization of the elbow, anterior posterior (AP) and lateral radiographs were taken to confirm concentric reduction. Posterior anterior (PA) and lateral radiographs of the wrist were also obtained intraoperatively to assess ulnar variance (radial length). Additionally, a lateral radiograph of the elbow was taken in full extension and supination to confirm the absence of ulnohumeral gapping or instability. 

Postoperative care

For all patients without diabetes, a perioperative corticosteroid protocol was utilized, as described by Desai et al. [4]. For patients receiving the corticosteroid protocol, we did not order additional medications for heterotopic ossification (HO) prophylaxis. Patients were placed in a long-arm posterior splint at 90° of flexion and neutral forearm rotation postoperatively. At two weeks, patients initiated active and passive elbow ROM under the supervision of a certified occupational hand therapist. For the initial cases in this series, the senior author utilized a hinged elbow brace after discontinuation of the postoperative splint. The hinged brace had a 30° extension block that was decreased by 10° per week, with brace removal by postoperative week 6. During the study period, this postoperative protocol was modified, and the senior author began using a brace-free, supine overhead ROM protocol, similar to that described by Schreiber et al. [18].

Statistics

Descriptive statistics were utilized for this investigation. Means, percentages and standard deviations, where appropriate, were reported throughout the manuscript and tables. 

## Results

There was a total of 24 operative TT cases treated during the study period. Three cases were excluded due to a follow-up period of <6 months, leaving a total of 21 cases (88%) available for analysis. Table 1 includes baseline and injury demographics for all included cases.

**Table 1:** Baseline and injury demographics for all included patients. SD, standard deviation; ASA, American Society of Anaesthesiologists; BMI, body mass index

Demographic variables	Value
Cases, (n)	21
Age in years, mean (SD)	54 (14)
Male, n(%)	12 (57%)
Dominant hand involved, n(%)	10 (48%)
Laterality right, n(%)	11 (52%)
BMI, mean (SD)	35 (9)
Active tobacco use, n(%)	4 (19%)
Diabetes, n(%)	2 (10%)
Rheumatoid arthritis, n(%)	0 (0%)
ASA rating, n (%)	
ASA 1-2	14 (66%)
ASA 3-4	7 (34%)
Mental, behavioral, or neurodevelopmental disorder, n(%)	4 (19%)
Currently employed, n(%)	10 (48%)
Married, n(%)	14 (66%)
Injury demographics	
Cases, (n)	21
High energy mechanism, n(%)	5 (24%)
Open injury, n(%)	0 (0%)
Radial head fracture classification, n(%)	
Type I	1 (5%)
Type II	4 (19%)
Type III	15 (71%)
Coronoid fracture classification, n(%)	
Type I	16 (76%)
Type II	5 (24%)
Type III	0 (0%)
Cases with associated ipsilateral upper-extremity injury, n (%)	5 (24%)
Associated injury type, n	
Scapula body fracture	1
Distal radius + scapula body fracture	1
Scaphoid fracture	3
Cases with any associated orthopedic injuries, n (%)	9 (43%)

The mean age was 54 years, and the dominant extremity was involved in 10 (48%) cases. Five cases (24%) had an associated ipsilateral upper-extremity injury. Table 2 includes descriptions of the surgical characteristics of the TT procedures. 

**Table 2:** Surgical characteristics for all included cases. SD, standard deviation; ORIF, open reduction, internal fixation

Case characteristics	Values
Cases, (n)	21
Operative time in minutes, mean (SD)	86 (18)
Radial head treatment, n(%)	
Fragment (<10%) excision	1 (5%)
ORIF	4 (19%)
Arthroplasty	16 (76%)
Coronoid fracture treatment, n(%)	
None	11 (58%)
Trans-osseous suture fixation	10 (48%)
Lateral ulnar collateral ligament treatment, n(%)	
Repair	9 (43%)
Repair with suture-tape augmentation	12 (57%)
Associated ipsilateral surgical procedures at time of index surgery, n(%)	6
Radius dorsal spanning plate	1 (17%)
Cubital tunnel decompression	1 (17%)
ORIF scaphoid fracture	2 (33%)
Repair common extensor/supinator origin	2 (33%)

**Figure 1:**
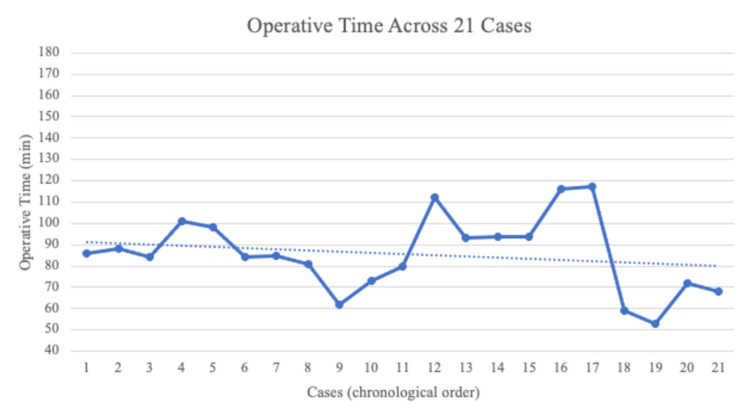
Chronological depiction of operative case lengths (in minutes) for the included TT procedures. TT, terrible triad

Sixteen cases (76%) underwent a metallic, cementless, radial head arthroplasty for a non-reconstruction radial head fracture. Of the 21 included cases, 10 underwent trans-osseous suture fixation of the fragment and anterior capsule. Nine cases (43%) underwent LCL repair whereas 12 cases (57%) had an LCL repair with suture-tape augmentation. The operative time averaged 89 min for the first 10 cases and 83 min for the subsequent 11 cases. Figure 1 chronologically depicts operative times for the included cases. 

Table 3 includes the outcomes (ROM and PROMs) as well as complications for the 21 included TT cases.

**Table 3:** PROMs, ROM, and complications for all included cases at a mean follow-up of 20 months postoperatively. SD, standard deviation; DASH, disabilities of the arm, shoulder, and hand; VAS, visual analog scale; PROMs, patient reported outcome measures; ROM, range of motion

Patient followup	Value
Cases, (n)	21
Months of follow-up	
Mean (SD)	20 (14)
Range	6-49
PROMs	
QuickDASH	
Mean (SD)	19 (14)
Range	0-45
VAS pain	
Mean (SD)	2.1 (2.4)
Range	0-8
ROM	
Flexion, mean (SD)	136° (11)
Extension, mean (SD)	-13° (9)
Flexion-Extension arc, mean (SD)	122° (17)
Cases with <100° flexion-extension arc, n(%)	2 (9%)
Pronation, mean (SD)	78° (9)
Supination, mean (SD)	68° (14)
Pronation-Supination arc, mean (SD)	145° (21)
Cases with <100° pronation-supination arc, n(%)	1 (5%)
Complications	
Cases, (n)	21
Cases with postoperative complication, n (%)	3 (14%)
Total postoperative complications, n	4
Instability / subluxation	3
Deep infection	1
Cases with any reoperation, n(%)	3 (14%)

The mean follow-up was 20 months and the mean QuickDASH was 19. For ROM, the mean flexion-extension arc was 122° and two cases (9%) had less than a 100° arc of motion. The mean pronation-supination arc was 145° at the time of final follow-up with one case (5%) having <100° arc of pronation-supination motion. The perioperative corticosteroid protocol was used in 19/21 (all cases without diabetes). 

**Figure 2:**
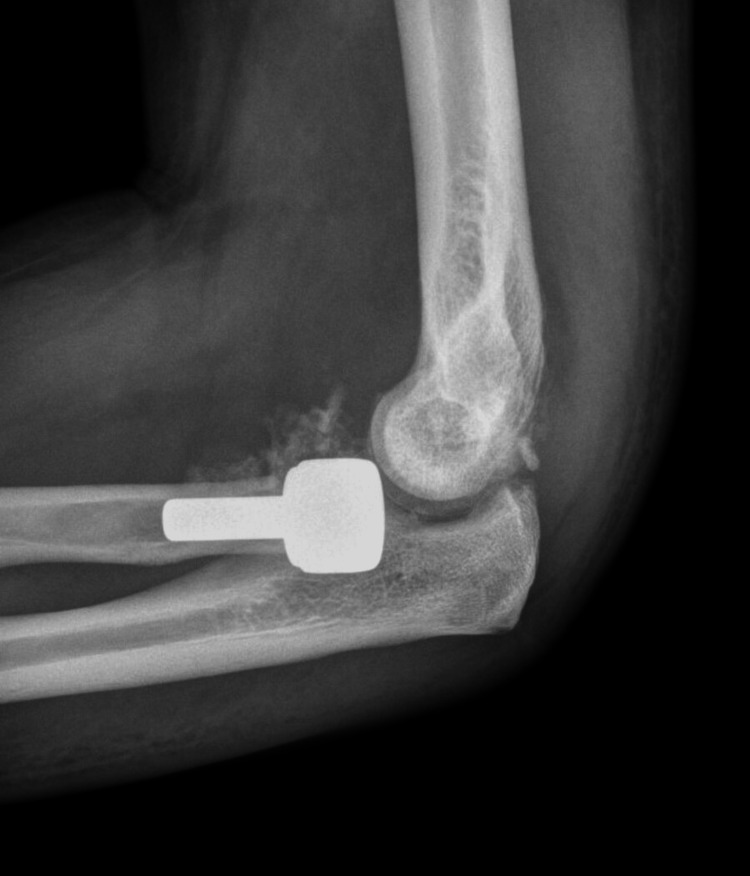
Lateral elbow radiograph demonstrating posterior subluxation of the radial head prosthesis 4 weeks after the index TT procedure. TT, terrible triad

Three cases (14%) had a postoperative complication, all of which underwent reoperation. Of the 21 included consecutive cases, these reoperations represented cases #1, #14, and #17 respectively. In the first case, deep infection and posterior subluxation of the radial head prosthesis were noted 4 weeks after the index procedure (Figure 2). 

The patient underwent debridement and irrigation, revision LCL reconstruction, and was treated with 6 weeks of IV antibiotics. The second case had early radiocapiteallar and ulnohumeral subluxation 3 weeks after radial head arthroplasty and LCL repair. This patient underwent open reduction, revision LCL reconstruction, and cross-pinning of his ulnohumeral joint for 3 weeks. In the third case, the patient had some instability of the radial head prosthesis 3 months after surgery. This patient underwent planned removal of the distal radius bridge plate 3 months after his index procedure, and the radial head prosthesis was removed at the same time. At the time of removal, the patient had a concentrically reduced ulnohumeral joint and no other procedures were performed. 

Table 4 includes a comparison of our patient demographics and results to other recently published series on the operative treatment of TT elbow injuries. 

**Table 4:** Comparison of outcomes reported in our series to recently published series on operative treatment of TT elbow injuries. NR, not reported; SD, standard deviation; ROM, range of motion; VAS, visual analog scale; DASH, disability of the arm, shoulder, and hand; MEPS, Mayo elbow performance score; ASES, American shoulder and elbow surgeon shoulder score; PROM, patient reported outcome measure; TT, terrible triad References: Ikemoto et al. (2017) [2], Toros et al. (2012) [19], Leigh and Ball (2012) [20], Zhang et al. (2014) [21], Giannicola et al. (2015) [22], Domos et al. (2018) [23], Liu et al. (2018) [5], Lee et al. (2019) [24]

Case variables	Our series	Ikemoto et al. (2017)	Toros et al. (2012)	Leigh and Ball (2012)	Zhang et al. (2014)	Giannicola et al. (2015)	Domos et al. (2018)	Liu et al. (2018)	Lee et al. (2019)
Cases (n)	21	21	16	24	21	26	22	42	24
Mean age	54	39	35	44	38	52	47	48	48
Operative time in minutes, mean	86	NR	NR	NR	NR	NR	NR	NR	151
Months follow-up, mean	20	31	35	41	32	31	32	31	30
ROM, mean°									
Flexion	136°	123°	133°	135°	136°	137°	134°	127°	138°
Extension	13°	22°	14°	8°	10°	10°	21°	20°	5°
Flexion-extension arc	122°	101°	119°	127°	126°	127°	113°	107°	128°
Pronation	78°	50°	72°	80°	70.5°	79°	73°	73°	81°
Supination	68°	65°	74°	75°	68.6°	77°	64°	71°	86°
Pronation-supination arc	146°	114°	146°	155°	139°	166°	137°	144°	167°
VAS Pain, mean	2	NR	NR	NR	NR	NR	2	NR	NR
Mean PROM and final follow-up	QuickDASH (19)	MEPS (84)	MEPS (95)	ASES (85)	MEPS (95)	QuickDASH (8)	QuickDASH(21)	MEPS (88)	MEPS (93)
Mean PROM and final follow-up	-	DASH (14)	DASH (9)	DASH (10)	-	-	-	-	-
Complication rate, %	19%	19%	16%	29%	23%	34%	41%	23%	16.7%
Reoperation rate, %	14%	38%	NR	25%	4.7%	23%	22%	9.5%	0%
Recurrent instability, %	14%	33%	NR	4%	0%	15%	NR	0%	0%
Deep infection, %	4%	4%	NR	4%	0%	NR	0%	0%	NR

## Discussion

The TT elbow fracture dislocations continue to represent a challenging clinical entity for both patients and surgeons. In aiming to define our early surgical experience treating these complex injuries, we found that functional outcomes, ROM, and surgical complications were comparable to other recently published series. This period of early learning while attempting to gain proficiency with a procedure has often been referred to as a “learning curve period” and has been variably defined by changes in operative time, outcomes, and complications [9, 11, 25-26]. In this consecutive series of 21 operatively treated TT elbow fracture dislocations performed at the start of clinical practice, we reported a mean QuickDASH of 19 with an average follow-up period of 20 months. Recent series assessing outcomes of operatively treated TTs have reported similar results with respect to the QuickDASH. With a mean follow-up period of over 2 years, Corbet et al. noted a mean QuickDASH of 16, Kim et al. reported a mean QuickDASH of 17, and Van Rysselberghe et al. reported a mean QuickDASH of 9 for operatively treated TTs [27-29]. A recent systematic review incorporating 16 studies reported a range of QuickDASH scores after TT surgery from 9 to 31 [30]. Similarly, postoperative ROM in our series appears consistent with recent investigations. Desai et al. defined the perioperative corticosteroid protocol utilized in our investigation [4]. Using this protocol, Desai et al. reported a mean flexion-extension arc of motion of 133° degrees, which compares to the mean flexion extension arc (122°) reported in our series. 

Prior investigations within upper-extremity surgery have aimed to define a “learning curve period” based on changes in operative times as a surgeon gains experience. In our series, operative times remained consistent throughout the study period and averaged 89 min for our first 10 cases and 83 min for our subsequent 11 cases. Operative time has been infrequently reported in prior TT series, however, prior shoulder arthroplasty studies have indicated decreases in operative times with increased case volumes [10, 25]. In contrast to our findings, Testa et al. found that higher case volumes decreased operative times for shoulder arthroplasty [10]. Outside of upper-extremity surgery, Konan et al. noted that operative times decreased by 46% after their first 10 hip arthroscopy cases in a single surgeon investigation [26].

We reported an overall reoperation rate of 14% in our series, with reoperations attributable to recurrent instability and deep infection. However, these complications were not limited to the early cases. Of the 21 included consecutive cases, reoperations occurred in cases #1, #14, and #17 respectively. Our overall complication and reoperation rates appear to be consistent with other recent TT series (Table 4). In a systematic review assessing 16 studies and 312 patients, Chen et al. reported a mean reoperation rate of 22% (range 0-55%) [30]. In another assessment of a single surgeon complication-based learning curve, Groh and Groh noted no differences in complications or reoperations relative to surgeon experience [25]. Similarly, Marti et al. performed a single-surgeon investigation of their initial 100 elbow arthroscopy cases and noted no evidence of increased complications during their learning curve period [11]. 

This investigation has a number of limitations that should be considered. There are inherent limitations associated with the retrospective design. Without a control group of cases performed by more senior or experienced surgeons, we relied on comparisons to recently published series and systematic reviews to compare outcomes, ROM, and complication rates, which is a less rigorous comparison than a control group. This investigation involved a single surgeon, which likely limits the generalizability of these findings. While we reported a mean follow-up period of 20 months, longer-term follow-up may have increased our reoperation rate secondary to late sequelae or post-traumatic conditions. 

## Conclusions

In conclusion, our investigation of a single fellowship-trained upper-extremity surgeon revealed no substantial learning curve with regard to operative time, surgical outcomes, or patient function. This may indicate that early-career upper-extremity surgeons at the start of clinical practice may be able to achieve outcomes similar to more experienced surgeons for operatively treated TT elbow fracture dislocations. There does not appear to be a substantial learning curve after fellowship training with respect to PROMs, complication rates, or operative time associated with surgical treatment of TT elbow injuries. Future investigations should aim to incorporate additional surgeons from multiple centers.
